# Genome sequences of Mx1, the first *Myxococcus* phage isolated, and Mx4, a generalized transducing myxophage

**DOI:** 10.1128/MRA.00904-23

**Published:** 2023-11-27

**Authors:** Alfonso López-Rojo, Simon A. Jackson, Diego Bernal-Bernal, Marisa Galbis-Martínez, Peter C. Fineran, Montserrat Elías-Arnanz

**Affiliations:** 1 Departamento de Genética y Microbiología, Área de Genética (Unidad asociada al IQFR-CSIC), Universidad de Murcia, Murcia, Spain; 2 Department of Microbiology and Immunology, University of Otago, Dunedin, New Zealand; 3 Genetics Otago, University of Otago, Dunedin, New Zealand; 4 Bioprotection Aotearoa, University of Otago, Dunedin, New Zealand; 5 Maurice Wilkins Centre for Molecular Biodiscovery, University of Otago, Dunedin, New Zealand; Queens College, Queens, New York, USA

**Keywords:** *Myxococcus xanthus*, myxophage, Mx1, Mx4, phage, Caudoviricetes, myxobacteria, Myxococcota

## Abstract

*Myxococcus xanthus* is the best-studied member of the phylum Myxococcota, but the bacteriophages infecting it and their characterization remain limited. Here, we present complete genomes of Mx1, the first *Myxococcus* phage isolated, and of an Mx4 derivative widely used for generalized transduction, both unclassified Caudoviricetes with long, contractile tails.

## ANNOUNCEMENT

The Gram-negative soil myxobacterium *Myxococcus xanthus* is a model organism to study bacterial multicellularity, motility, social behavior, light response, predation, and biosynthesis of lipids, steroids, and secondary metabolites (1–6). Its large genome contains three CRISPR-Cas systems (7, 8), and the regulation of two of these has been examined (7, 9, 10). However, their role in phage defense remains unexplored. Moreover, few myxophages have been isolated (11), with genomes only available for the lysogenic Mx8 (GenBank accession no. AF396866.1) and Mx9 (GenBank accession no. OQ709411.1) (12) and the lytic Mx4 (GenBank accession no. OK085710.1) (13). Here, we report whole-genome sequences of two unclassified Caudoviricetes: Mx1, the first myxophage isolated (14, 15), and the Mx4-derivative Mx4 *ts27htf-1hrm-1*, most commonly used for generalized transduction (16). Mx4 *ts27htf-1hrm-1* was generated from a cross between Mx4 *ts18ts27htf-*1, a double temperature sensitive (26°C–28°C, permissive; 33°C–35°C, restrictive) and high-frequency transduction mutant (obtained by sequential nitrosoguanidine treatments), and Mx4 *hrm-1*, a host range mutant (obtained spontaneously) that can infect wild-type *M. xanthus* strains (17, 18).

Phage stocks were obtained using *M. xanthus* strain DK1050 (8) as host, following standard protocols (13, 14, 17). Phage DNA was purified from 0.5 mL of lysate using a Qiagen DNeasy Kit, and then Illumina Nextera XT sequencing libraries were constructed (19). Illumina MiSeq 150 bp paired-end sequencing was performed (19), and raw demultiplexed reads (Mx1, 211,070 reads; Mx4, 235,764 reads) were trimmed and quality filtered using trimommatic v0.39 (20). The genomes were assembled using the Geneious (2019) *de novo* assembler, resulting in closed circular contigs for both phage genomes. The coverage of the final assemblies (327 × Mx1; 247 × Mx4 *ts27htf-1hrm-1*) was determined by mapping the input reads to the final assemblies using Bowtie2 (21). An automated first annotation of the genomes using RAST (22) was further refined with BLAST (23), InterProScan (https://www.ebi.ac.uk/interpro), HHPred (24), a jackhmmer search against GenBank phage proteins using HMMER (25), tRNAscan-SE (26), and ARAGORN (27).

The 169,621-bp Mx1 genome has 55.5% GC content, compared to 70.1% for the 56,975-bp Mx4 *ts27htf-1hrm-1* and 68.8% for the *M. xanthus* host. Mx1 contains 250 putative coding DNA sequences (CDSs), 86 located on the positive strand and 164 on the negative strand, but most CDSs (187) exhibited no homology to characterized proteins or domains, and 22 tRNA genes were annotated. The genome start point was assigned to the first of several CDSs oriented in the same direction in a region containing the putative large terminase gene. Ninety CDSs were annotated in the 56,975 bp Mx4 *ts27htf-1hrm-1* genome, which is 99.96% identical to that of Mx4, but differs in 21 substitutions, 15 of which are non-synonymous; the sequenced lysate population was also heterozygous at position 39,140 (A/G; 57%/43%). Eight variants distributed among four predicted structural genes may account for the phenotypical differences between Mx4 and its derivative. In conclusion, the Mx1 genome is larger than Mx4, has a considerably lower GC content than Mx4 or the host, and encodes 22 tRNA genes (Mx4 encodes none), suggesting a longer period of Mx4 coevolution with its bacterial host.

## Data Availability

Raw sequencing read data on the genome sequences of phages Mx1 and Mx4 *ts27htf-1hrm-1* are deposited at NCBI under BioProject accession number PRJNA901695, and their genome sequences are under accession numbers OR576727 (Mx1) and OR576728 (Mx4 *ts27htf-1hrm-1*).
